# Patient perception of understanding health education and instructions has moderating effect on glycemic control

**DOI:** 10.1186/1471-2458-14-683

**Published:** 2014-07-04

**Authors:** Gin-Den Chen, Chien-Ning Huang, Yi-Sun Yang, Chin-Yin Lew-Ting

**Affiliations:** 1 Department of Public Health and Institute of Health Policy and Management, College of Public Health, National Taiwan University, No. 17, Xu-Zhou Road, 100 Taipei, Taiwan; 2 Department of Obstetrics and Gynecology, Chung Shan Medical University Hospital, No. 110, Chien-Kuo N Road, Section1, 402 Taichung, Taiwan; 3 School of Medicine, Chung Shan Medical University, No. 110, Chien-Kuo N Road, Section1, 402 Taichung, Taiwan; 4 Department of Metabolism, Chung Shan Medical University Hospital, No. 110, Chien-Kuo N Road, Section1, 402 Taichung, Taiwan

**Keywords:** Health literacy, Glycosylated hemoglobin, HbA1c, Type 2 diabetes mellitus, DM, Glycemic control

## Abstract

**Background:**

Whether health literacy is independently associated with processes or outcomes of diabetes-related care is controversial. We tried to demonstrate the interaction of health literacy and understanding of health education and instructions in achieving glycemic control.

**Methods:**

Five hundred and one consecutive patients with type 2 diabetes mellitus (DM) in the outpatient clinic of the metabolism department were recruited into this pilot study. The demographic data were collected from patients’ self-reports. The clinical background information was collected through electronic medical records. A questionnaire derived from part of the Mandarin Health Literacy Scale was used to measure numeracy and functional health literacy of people with diabetes. Health literacy levels were categorized into inadequate, marginal and adequate. Patient self-ratings of their perceived understanding of the health education information and instructions provided by their case manager in the past were categorized into two subgroups: better and poor. Patients with an HbA1c level equal to or below 7% were considered to have good glycemic control. Multivariate logistic regression was used to find associated factors of health literacy and understanding of health education and instructions. GENMOD procedures were used to analyze repeated outcome measurements of glycemic control.

**Results:**

Higher educational attainment and higher household income (odds ratios were 2.23 and 2.22, respectively) were significantly associated with patients who had adequate health literacy. Higher educational attainment and patients with a family history of DM (odds ratios were 4.99 and 1.85, respectively) were significantly associated with better understanding of health education and instructions. Adequate health literacy is not the only factor associated with good glycemic control. The effect of adequate health literacy in achieving good glycemic control might be masked by patients with better understanding of health education and instructions.

**Conclusions:**

Our results revealed that not only were patients with adequate health literacy associated with good glycemic control but patients with marginal health literacy were also able to achieve good glycemic control. Adequate health literacy and better understanding of health education is highly correlated. The role of adequate health literacy on glycemic control could be suppressed if variables are over-controlled during analysis.

## Background

In the past two decades, health literacy as a discrete form of literacy, different from general literacy, has become increasingly important for social, economic and health development. Improvement in the health literacy of a community would complement efforts to improve health services and disease control [1]. Health literacy is a set of skills, including functional, interactive, numeracy and critical skills, which people need to function effectively in the health care system. These skills include the ability to read and understand written text, locate and interpret information in documents, write or complete forms; speak and listen effectively and communicate about health-related information; navigate the health care system and make appropriate health decisions; and use numeric information for tasks, such as interpreting medication dosages, food labels, and blood glucose measurements [2].

“Inadequate health literacy poses a major barrier to caring for or educating patients with chronic diseases” such as hypertension or diabetes (i.e. lack knowledge of their disease, important lifestyle modifications, interpreting medication dosages, food labels, blood glucose measurements and essential self-management) [3]. Al Sayah et al. conducted a systematic review that addressed the association between health literacy and health outcomes in diabetes. They found that the majority of the health literacy studies (87.5%) were from US primary care settings. Low health literacy is consistently associated with poorer diabetes knowledge. However, there is little sufficient or consistent evidence suggesting that health literacy is independently associated with processes or outcomes of diabetes-related care. Based on their results, they concluded that “it may be premature to routinely screen for low health literacy as a means for improving diabetes-related outcomes” [4].

Currently, health literacy in Taiwan is not well evaluated. In 2010, Lee and colleagues conducted a nationwide survey of health literacy, health status, and healthcare utilization of Taiwanese adults using the Mandarin Health Literacy Scale (MHLS). They found that low (inadequate and marginal) health literacy is prevalent in Taiwan, but health literacy is not a predictor for health care utilization (according to participants’ self-reports) among Taiwanese adults [5]. Taiwan’s health care system today is encountering pressure of escalating health care expenditures, an increase in health services utilization as well as a rapidly aging population [6]. The prevalence of diabetes mellitus (DM) in Taiwan has increased to 8% according to a National Health Insurance (NHI) report [7]. The direct cost of diabetes care is up to 4% of the annual expenditure of the NHI which does not include the expenditure associated with diabetes-related complications. In contrast to the development and progress in the health service system in Taiwan, poor glycemic control is prevalent. Inadequate health literacy may impact self-management and engagement in health promotion and thus be a predisposing factor for the poor glycemic control. However, this hypothesis needs scientific data for clarification.

We hypothesized that patients with diabetes with adequate health literacy would lead to good glycemic control, i.e. lower glycosylated hemoglobin (HbA1c) equal to or below 7%. We expected patient perception of understanding health education and instructions provided by the case manager to be associated with glycemic control. In this study, we evaluated health literacy in patients with diabetes and also looked at patients’ self-ratings of their understanding of health education and instructions provided by their case manager. Further, we also looked for an interaction between patient perceived understanding health education and instructions in controlling DM and health literacy.

## Methods

### Study population and setting

This study was conducted at Chung Shan Medical University Hospital in central Taiwan for patients with type 2 diabetes in the outpatient clinic of the metabolism department. Patients in this outpatient clinic were informed of the purpose and procedures of the study and were invited to participate. Written consent was obtained. Patients with poor vision who were unable to see written items on the questionnaire, patients with cognitive problems, or disabled due to cerebral vascular accident, or in poor physical condition were excluded from the study. In order to avoid selection bias, 501 consecutive patients were recruited into this study after consultation with the same diabetic specialist and case manager, from August 2012 to December 2012. In the diabetic clinic, the case manager performed counseling including health education and instructions for medications and diet control based on the current Chronic Care Model in Taiwan. This care model has been initiated by the Taiwanese Association of Diabetes Educators and was designed to provide health information and empower patients in the self-management of glycemic control and associated chronic diseases.

We obtained information in two steps. First, patients were asked to answer a questionnaire which included demographic data, basic clinical background, current health status, and a self-rating by patients who thought they understood the health education information and instructions provided by their case manager in the past. Second, they were asked to read two diabetes-related reading comprehension sheets and then answer 11 questions attached to the reading sheets regarding information on overweight, body mass index and hypoglycemic agents. The study protocol and questionnaire content was approved by the Institution of Review Board of Chung Shan Medical University Hospital.

### Demographic data and clinical characteristics

The demographic data in the questionnaire including age, gender, educational attainment, and household income were collected from the patients’ self-reports. The clinical background information including duration of DM, family history of DM, regimens for glycemic control, original and most recently measured HbA1c, body mass index, diabetes-related complications and comorbidities were collected through electronic medical records. The trained research assistant helped those who were illiterate or had very poor literacy to complete this section of the questionnaire. Patients’ diabetes-related complications were grouped as peripheral neuropathy, retinopathy, renal failure, and macroangiopathy. Their comorbidities were categorized as cardiovascular disease, hypertension and hyperlipidemia, cancers, cerebrovascular accidents (stroke episode), and others (less than 3% in these patients) for analysis after checking their frequency distribution. Further, we dichotomized these patients with or without comorbidities and complications for analysis.

Originally, educational attainment was classified as illiterate, compulsory school, senior high school, and college or more and was then further re-grouped into two categories (i.e. equal to or lower than compulsory school which is less than 9 years of schooling and higher than compulsory school) for analysis. Six options were given for household incomes which were then grouped into two categories (lower household income or above average) based on the self-reported household incomes distribution (from < $200.000 NTD per year to > $1,000,000 NTD per year; NTD: new Taiwan dollar; 30 NTD≒1 USD) for data analysis. Educational attainment and household income were used separately to represent the socioeconomic levels.

Patients who thought they understood the health education information and instructions provided by their case manager in the past were categorized into five levels (Likert scale). Then we re-categorized patients’ self-rated levels into two subgroups for analysis. Patients who thought they understood all or most of the health education information and instructions provided by their case manager were re-categorized as the “better understanding” subgroup. The others who thought they understood half or less of the health information and instructions were re-categorized as the “poor understanding” subgroup.

### Diabetes-related health literacy

The two diabetes-related reading comprehension sheets attached to the questionnaire were taken from the Mandarin Health Literacy Scale (MHLS). Originally, the MHLS was developed and validated by Kuo et al. in 2010 to evaluate the generic health literacy of Mandarin Chinese writing and speaking adults in Taiwan with the full text in Mandarin Chinese [8]. The two domains derived from the MHLS focus on the basic knowledge of body weight control and medication information about hypoglycemic agents in this study. We used them in this study to measure patients’ functional and numeracy health literacy.

After the patients read the written sheets, they were asked to answer 11 questions from the MHLS which were designed to measure the ability to understand health information about body weight control (four items; including abdominal circumstances, criteria for being overweight, knowing what body mass index is and how to calculate body mass index) and information about hypoglycemic agents on the prescription labels/instructions (seven items; including name of the patient, doses of medications, time to take medications, interval of taking medication, prescription length, adverse effects of medications, avoiding immediate activities after taking medications). Patients could access, locate and interpret the information from the written sheets to answer the questions. These 11 questions were answered by the patients without any assistance.

The health literacy levels derived from the two domains of the MHLS were grouped into three categories: inadequate, marginal, and adequate, based on referral cutoffs of the full MHLS version validated with Taiwanese adults [9]. The cut-off points of diabetes-related health literacy were set as follows: Patients with fewer than 6 correct answers were categorized as inadequate health literacy (18%, 84/467). One hundred and thirty-seven patients (29.3%) with 6 to 9 correct answers were categorized as marginal health literacy. Patients (52.7%, 246/467) with 10 or 11 correct answers were categorized as adequate health literacy. We assessed the association between these three subgroups of health literacy levels and glycemic control outcomes. We also assessed the factors associated with adequate health literacy and better perception of understanding health education and instructions provided by their case manager in the past.

### Outcomes measurements

The HbA1c levels were checked every three months after treatment. Patients with an HbA1c level equal to or below 7% were considered to have good glycemic control (goal HbA1c level as suggested by consensus of Taiwan National Health Insurance). The most recent HbA1c levels were used as main indicators of the current glycemic control outcome. The initial HbA1c levels (checked immediately after the patient was diagnosed with type 2 DM) were used as baseline, and all the HbA1c levels were also collected from the electronic medical records. We used the average HbA1c level from the previous year (before the most recent HbA1c) and the most recent HbA1c level as outcomes of repeated measurements. The HbA1c levels (7% as a cut-off point) were dichotomized as a binary variable for analysis.

### Statistical analysis

Descriptive statistics (mean, standard deviation or percentage) were performed to examine the level of diabetic-related health literacy and outcomes of glycemic control in the samples by socio-demographic attributes, clinical characteristics, and self-rated understanding of health education and instructions. Significant differences in different levels of health literacy were evaluated using chi-square test, Student *t* test, or one-way analysis of variance (ANOVA). Scheffe’s multiple-comparison procedure was used for post hoc check. Alpha level was set as 0.05.

We checked whether patient perception of understanding health education and instructions provided by their case manager in the past had an interaction with health literacy on glycemic control or a likely multicollinearity effect on patients with adequate health literacy and perception of understanding health education and instructions provided by their case manager in the past. We used health literacy and better perceived understanding of health education and instructions as independent variables and HbA1C as a dependent variable to perform multiple regression analysis (data not shown). The variance inflation factors were 1.08 and 1.8, respectively (none of the variance inflation factor were > 10). The Pearson correlation coefficient was 0.17 for HbA1C and better understanding of health education and instructions (P < 0.05) as well as 0.27 for health literacy and better understanding of health education and instructions (P < 0.05). These results revealed that patient perception of understanding health education and instructions is highly correlated with health literacy and is not due to a multicollinearity effect. Therefore, these two variables were used in Hierarchical regression models to check their interactive effect.

The demographic, socioeconomic and clinical factors that were associated with patients’ better health literacy and better understanding of health education and instructions were analyzed using multivariate logistic regression analysis. Factors associated with the outcome of glycemic control were analyzed using the GENMOD Procedures for analysis of generalized estimating equation (GEE) parameter estimates. All statistical analyses were performed using the Statistical Analysis Software package, SAS version 9.3.

## Results

A total of 501 patients at the diabetic outpatient clinic were invited to participate in this survey. Thirty-four patients (6.8%; 16 rushed for time, 12 privacy reason, and 6 prior appointments) refused to answer the questionnaire. Forty-four (9.4%) patients who were illiterate or had poor literacy completed two portions of the questionnaire (demographic data and clinical characteristics) with the help of the research assistant were also included in the data analysis. They were categorized as having inadequate health literacy because they could not answer the diabetes-related reading comprehension sheets (they had no correct answers). In total, data from 467 patients were used in the analysis. The response rate in this study was 93.2%. The socio-demographic data, clinical characteristics, and self-rated perception of understanding health education and instructions are shown in Table 1.

**Table 1:** Demographic and clinical characteristics of all patients with Type 2 diabetes mellitus (DM) (N = 467) categorized by level of Health literacy (HL)

	**Total, n = 467**	**Adequate, n = 246(%)**	**Marginal, n = 137(%)**	**Inadequate, n = 84(%)**
Age, Mean (S.D.)	61.4(10.4)	58.9(11.1)^*†^	61.7(8.5)^*‡^	68.3(7.4)^†‡^
Gender, Female	255(54.6)	115(46.8)^*†^	81(59.1)^*‡^	59(70.2)^†‡^
Education > compulsory school	180(38.5)	129(53.4)^*†^	45(32.8)^*‡^	6(7.1)^†‡^
Household income above average	226(48.4)	153(62.2)^*†^	61(44.5)^*^	12(14.3)^†^
Duration of DM, years (S.D)	9.3(7.3)	8.7(7.0)^†^	9.1(7.3)	11.3(7.5)^†^
With family history of DM	285(61.0)	157(63.8)^†^	88(64.2)^‡^	40(47.6)^†‡^
With complications	224(52.6)	111(50.9)	64(50.4)	49(60.5)
With comorbidities	376(88.5)	188(86.2)	117(92.1)	71(88.8)
Initial HbA1c%, Mean (S.D.)	9.1(2.2)	9.0(2.1)^†^	8.8(2.2)^‡^	9.6(2.5)^†‡^
Current HbA1c%, Mean (S.D.)	7.8(1.6)	7.8(1.6)	7.8(1.7)	7.8(1.5)
Medications for controlling DM				
Hypoglycemia agents	366(78.4)	198(80.5)	110(80.3)	58(69.0)
Insulin	40(8.6)	21(8.5)	7(5.2)	12(14.3)
Hypoglycemia agents and insulin	61(13.1)	27(11.0)	20(14.6)	14(16.7)
Better understanding of health education and instruction	375(80.3)	220(89.4)^*†^	103(75.2)^*‡^	52(61.9)^†‡^

S.D. standard deviation.

Adequate adequate health literacy.

Marginal marginal level of health literacy.

Inadequate inadequate level of health literacy.

With family history of DM patients have a positive family history of DM.

With complications patients with any diabetic complications.

With comorbidities patients with other comorbidities except DM.

*significant difference for analyzing adequate health literacy vs. marginal health literacy.

†significant difference for analyzing adequate health literacy vs. inadequate health literacy.

‡significant difference for analyzing marginal health literacy vs. inadequate health literacy.

ANOVA for age, duration of diabetes mellitus, first time and current body weight, first time and current body mass index, initial HbA1c, current HbA1c and regimens for control DM; post hoc test by Scheffe’s multiple comparison procedure.

Chi square for analyzing gender difference; *, †, ‡ p < 0.05.

There were statistically significant differences among the subgroups of inadequate, marginal and adequate health literacy with respect to age, gender distributions, duration of DM, educational attainment, household income, and self-rated understanding of health education and instructions (Table 1). There were no significant differences in medications for controlling DM associated with the different literacy levels or glycemic control.

Higher educational attainment and higher household income (odds ratios were 2.23 and 2.22, respectively) were significantly associated with patients with adequate health literacy. Advanced age (odds ratio was 0.97) had a negative association with adequate health literacy (shown in the second column of Table 2). In the fourth column of Table 2, educational attainment higher than compulsory school and patients with a family history of DM (odds ratios were 4.99 and 1.85, respectively) were significantly associated with better perceived understanding of health education and instructions provided by their diabetic specialist and case manager.

**Table 2:** The Logistic regression for evaluating the demographic, socioeconomic and clinical factors which might be associated with adequate health literacy and better understanding of health education and instructions

	**Adequate health literacy**		**Better understanding of health education and instructions**	
	O.R.	95% of C.I.	O.R.	95% of C.I.
Age	0.97^*^	0.95-0.99	0.97	0.94-1.00
Gender, Female	0.67	0.44-1.03	0.74	0.44-1.27
Educational level > Compulsory school	2.23^*^	1.39-3.56	4.99^*^	2.43-10.27
Household income above average	2.22^*^	1.41-3.48	0.61	0.35-1.09
With family history of DM	1.09	0.71-1.69	1.85^*^	1.10-3.11
Without comorbidities	1.03	0.52-2.04	0.73	0.33-1.63
Without complications	0.82	0.53-1.27	0.83	0.49-1.41

DM diabetes mellitus.

O.R. odds ratio;

With family history of DM patients have a positive family history of DM.

With complications patients with any diabetic complications.

With comorbidities patients with other comorbidities except DM.

95% C.I. 95% confidence intervals.

^*^P <0.05.

The GENMOD procedure for analysis of parameter estimates was used to evaluate the outcomes by repeated measurements (Table 3). In model 1, we checked the role of health literacy in glycemic control. We found that adequate and marginal health literacy levels were significantly associated with good glycemic control outcomes (coefficient for marginal and adequate levels were 0.69 and 0.57, respectively). In model 2, we found that patients with marginal health literacy and better perceived understanding of health education and instructions were significantly associated with good glycemic outcomes (coefficient: 0.62 and 0.46; 95% confidence limits: 0.11 ~ 1.14 and 0.01 ~ 0.91; respectively). However, the effect of adequate health literacy was not significantly associated with glycemic outcomes (coefficient: 0.46; 95% confidence limits: -0.03 ~ 0.95).

**Table 3:** **The GENMOD Procedures for evaluating the contributing factors associated with good glycemic control (HbA1c ≦7**%**)**

		**Model 1**		**Model 2**		**Model 3**		**Model 4**		**Model 5**
	**β**	**95% of C.L.**	**β**	**95% of C.L.**	**β**	**95% of C.L.**	**β**	**95% of C.L.**	**β**	**95% of C.L.**
**Intercept**	-1.41	-1.8 ~ -1.00	-1.71	-2.20 ~ -1.23	-2.17	-2.97 ~ -1.37	-2.65	-4.00 ~ -1.30	2.04	0.11 ~ 3.97
**Health Literacy**, 2 vs.1	0.69*	0.17 ~ 1.20	0.62*	0.10 ~ 1.14	1.32*	0.32 ~ 2.32	0.69*	0.17 ~ 1.22	0.19	-0.34 ~ 0.73
**Health Literacy**, 3 vs.1	0.57*	0.09 ~ 1.05	0.46	-0.03 ~ 0.95	0.94	-0.12 ~ 1.99	0.54*	0.02 ~ 1.06	0.26	-0.26 ~ 0.78
**Better-understanding**			0.46*	0.01 ~ 0.91	1.09*	0.16 ~ 2.03	0.39	-0.07 ~ 0.85	0.44	-0.04 ~ 0.92
**Socio-demography**										
Age							0.02	-0.01 ~ 0.03	0.01	-0.01 ~ 0.03
Gender, Female							-0.17	-0.52 ~ 0.18	-0.10	-0.44 ~ 0.25
Education > Compulsory school							0.24	-0.15 ~ 0.63	0.07	-0.29 ~ 0.43
Household income above average							-0.10	-0.49 ~ 0.29	-0.05	-0.42 ~ 0.32
**Clinical features**										
Initial HbA1c levels									-0.48*	-0.62 ~ -0.34
Duration of DM									-0.02	-0.05 ~ 0.01
With family history of DM									0.27	-0.09 ~ 0.64
Without comorbidities									-0.23	-0.83 ~ 0.36
Without complications									0.33	-0.01 ~ 0.67
**Interaction of health literacy and better understanding**										
Better understanding × marginal health literacy					-0.95	-2.12 ~ 0.22				
Better understanding × adequate 2health literacy					-0.66	-1.84 ~ 0.53				

Health Literacy, 2 vs. 1 Marginal health literacy vs. inadequate health literacy.

Health Literacy, 3 vs. 1 Adequate health literacy vs. inadequate health literacy.

Better-understanding patients thought they understood all or most of the health education information and instructions provided by their case manager; reference group was poor understanding group.

With family history of DM patients have a positive family history of DM.

With complications patients with any diabetic complications.

With comorbidities patients with other comorbidities except DM.

β coefficient.

95% C.L. 95% confidence limits.

*P < 0.05.

We checked whether patient perception of understanding health education and instructions had an interactive effect on health literacy in glycemic control in model 3. The interactive terms of health literacy and better perceived understanding of health education and instructions showed no significant difference (coefficient: -0.95 and -0.66 for better understanding of health education and instructions versus marginal and adequate level of health literacy, respectively). The association of adequate health literacy in achieving good glycemic control was masked by patients with better perceived understanding of health education and instructions. This phenomenon might be caused by high correlations between better perceived understanding of health education and instruction and health literacy especially in the adequate health literacy subgroup.

The association of adequate and marginal health literacy was slightly enhanced (compared with those in model 2), but the association of better understanding of health education and instructions had no significant difference after demographic and socio-economic factors were added as controlling variables in model 4. In model 5, we added demographic, socio-economic factors and their clinical features as control variables and only the lower initial HbA1c level was associated with good current glycemic control outcomes (coefficient was -0.48, 95% confidence limits were -0.62 ~ -0.34). Not only the association of health literacy but also the association of patient understanding of health education and instructions were suppressed in glycemic control if these variables were controlled.

## Discussion

The current study reveals that adequate health literacy is not the only factor associated with good glycemic control for these patients. Patients with marginal levels of health literacy are also able to achieve good glycemic control if they can understand health education and instructions. Adequate health literacy and better understanding of health education are highly correlated. The association of adequate health literacy in achieving good glycemic control might be masked by patients with better perceived understanding of health education and instructions. The association of health literacy with glycemic control could be suppressed if variables are over-controlled during analysis.

The distribution of health literacy among our respondents in three subgroups (inadequate health literacy: 18.0%; marginal or ordinary health literacy: 29.8%; adequate health literacy: 52.2%) was similar to Paasche-Orlow et al. [10]. They used pooled analyses from a systematic review to show that the prevalence of low health literacy was 26% (95% confidence interval, 22% to 29%) and marginal health literacy was 20% (95% confidence interval, 16% to 23%). They found that low health literacy was associated with level of education, ethnicity, and age. A study conducted in Japan by Ishikawa et al. also found that advanced age, lower educational attainment, and lower economic status among their diabetic patients tended to be associated with lower health literacy [11]. However, a report from Jeppesen et al. revealed that diabetes patients with limited health literacy had a lower education level, were male and advanced in age [12]. Our results showed that the inadequate and marginal subgroups had more females, patients advanced in age, longer duration of DM, lower educational attainment, and lower household income compared to the adequate health literacy subgroup (Table 1). We had more females in the inadequate and marginal subgroups which might be a sampling bias due to the collection of consecutive patients from the outpatient clinic during the study interval.

Al Sayah et al. found a positive association between health literacy and understanding health education as well as diabetes knowledge [4]. In this study, we also discovered that more patients in the adequate and marginal subgroups thought they understood health education and instructions better than patients in the inadequate subgroup (89.4% of adequate health literacy subgroup, 75.2% of marginal level of health literacy subgroup and 61.9% of inadequate health literacy subgroup; all p < 0.05, Table 1). These findings are similar to the results of Williams et al. [3]. They conclude that “inadequate functional health literacy poses a major barrier to educating patients with the symptoms of hypoglycemia because they have less knowledge of their disease”.

In the multivariate logistic regression model, we found that advanced age is a negative factor associated with adequate health literacy. Patients with a higher socioeconomic status (higher educational attainment and higher household income) were significantly associated with having adequate health literacy (Table 2). These findings are similar to the results reported by Lee et al. where the full version of the MHLS was used to conduct a national survey of Taiwanese adults in 2008 [5]. However, our findings also revealed that the patients with higher educational attainment (higher than compulsory school) and family history of DM had a significant association with patient perception of understanding health education and instructions provided by their diabetic specialist and case manager in the past. Osborn et al. reported that health literacy is not directly associated with self-care and HbA1c but is indirectly associated with better outcomes through social support [13]. However, our findings revealed that higher educational attainment was associated with patients’ perceived understanding of health education and instructions (odds ratios: 4.99, Table 2). The roles of higher educational attainment in general literacy, health literacy and glycemic control needs to be further clarified in the future.

In our study, half of our patients with DM were elderly and their mean duration of DM was 9.3 years. Based on our findings, an increase in patient understanding of health education and instructions on glycemic control procedures as well as support by care providers seemed to be very important in helping our patients achieve diabetic control because patients in the marginal and inadequate health literacy subgroups were older than patients in the adequate health literacy subgroup. Overland et al. recommended that efforts to improve patients’ diabetes outcomes should focus on how to reduce literacy demands of health literature (or instructions and education provided by case managers) to improve patient comprehension [14], especially among patients with inadequate health literacy and to effectively deliver health education and instructions to patients.

The role of health literacy in glycemic control seems to be controversial. Morris et al. indicated that “functional health literacy, as measured by the Short Test of Functional Health Literacy for Adults, is not associated with glycated hemoglobin and self-reported diabetes complications in a cross-sectional study of older adults with diabetes under relatively good glycemic control” [15]. Schillinger et al. conducted a cross-sectional observational study in two primary care clinics of a university-affiliated public hospital in San Francisco and revealed that patients with inadequate health literacy, compared to patients with adequate health literacy, were less likely to achieve tight glycemic control (HbA1c ≦ 7.2%) and were more likely to have poor glycemic control (HbA1c ≧ 9.5%) and to report having retinopathy [16]. Cavanaugh et al. found low diabetes-related numeracy skills were possibly associated with poorer glycemic control. However, no direct relationship was observed between health literacy and glycemic control [17]. Some researchers have noticed that health literacy might have an indirect effect on diabetes self-care and glycemic control through its association with social support [13]. Different measurements of health literacy and study samples would influence the interpretation of the effect of health literacy on glycemic control. The relationships between health literacy and perceived understanding of health education and instructions also need further exploration. When we controlled the interaction of health literacy levels and better perceived understanding of health education and instructions in our regression model, coefficients of interaction terms between health literacy levels and better perceived understanding of health education and instructions were negative, but there were no significant differences in model 3. The association of marginal health literacy and better perceived understanding of health education and instructions with glycemic control was significantly enhanced. The association of adequate health literacy with glycemic control was also enhanced but not significantly. Our results suggest that the association of perceived understanding of health education and instructions with glycemic control is not consistent in patients with different health literacy levels.

The mechanisms underlying the relationships between health literacy and glycemic control are also unclear. Schillinger et al. used the same patient source to determine relationships between educational attainment and health literacy in glycemic control and reported that health literacy mediated the relationship between education and glycemic control in a low-income population by using a path analytic model [18]. We measured the roles of health literacy levels and better perceived understanding of health education and instructions play in glycemic control using the GENMOD procedure to check which factors are associated with glycemic control. Marginal health literacy and patients who thought they understood most of the health education and instructions were significantly associated with patients could achieve their glycemic control goal in model 2 of Table 3.

Our study showed that not only adequate health literacy is associated with good glycemic control but patients with marginal health literacy are also associated with good glycemic control in model 1 of Table 3. However, adequate health literacy had no significant association with glycemic control after better understanding of health education and instructions was added to the regression model (model 2). The role of health literacy became ambiguous when demographic, socioeconomic, and clinical factors such as comorbidities and complications were added to the regression model as control variables (model 4 and 5 in Table 3). These unique findings may be a result of the high correlation between understanding of health education and health literacy especially in the adequate health literacy subgroup. These scenarios in the statistical analysis in Table 3 might be due to over-control of variables causing an inconclusive role of health literacy in glycemic control of diabetes. The potential reason was that we put highly correlated variables in regression model. In a systematic review by Al Sayah et al. [4], on the role of health literacy in individuals with diabetes they concluded that “the discrepant results of the relationship between health literacy and several health outcomes in people with diabetes might be caused by over-adjustment of potential confounders” [4].

There are several limitations in this study. First, this study was conducted in the outpatient clinic of the metabolism department in the same hospital so selection bias could not be completely avoided. Our patient sample had more female patients. Second, 9.4% of the patients were classified as illiterate which is higher than the percentage reported by the Taiwan Ministry of the Interior reported in 2013 [19]. The high illiteracy rate in our patient sample seems to be inconsistent with that report. The adult literacy rate is 98.29%. However, in our study, the mean age of our patient sample was 61.4 years. Most of the illiterate were found to be elderly (86.6%) and illiteracy among females was higher than among males. Third, the cutoff points of the subgroups of health literacy in this study were based on the proportional distributions of the full MHLS version validated with Taiwanese adults [5]. This was not an arbitrary decision. Therefore, the criteria for health literacy levels might be difficult to be generalized to other studies. Fourth, this was a cross-sectional study. The timings of initial HbA1c levels were different for each patient. The initial HbA1c levels were collected from electronic medical records after patients had completed the questionnaire. In order to reduce potential information bias, we used the average of HbA1c levels from previous year (before the most recent HbA1c) as one of the outcomes for repeated measurements in our statistical model.

## Conclusion

Not only were patients with adequate health literacy associated with good glycemic control but patients with marginal health literacy were also able to achieve good glycemic control. Patient perception of understanding health education and health literacy levels is highly correlated. Taken together, the regression model indicates an inconclusive role of health literacy in this study. The effect of health literacy on glycemic control could be suppressed if variables are over-controlled during analysis.

## Competing interests

The authors declare that they have no competing interests.

## Authors’ contributions

GDC analyzed research data, wrote drafts and revised the manuscript. CYLT contributed to the discussion and reviewed the manuscript. CNH designed the study protocol and provided patients sources. YJY designed the study protocol and revised the manuscript. All authors read and approved the final manuscript.

## Pre-publication history

The pre-publication history for this paper can be accessed here:

http://www.biomedcentral.com/1471-2458/14/683/prepub
